# SPECIAL FOCUS FACILITIES AND COVID-19: THE IMPACT ON STAFF AND RESIDENT HEALTH

**DOI:** 10.1093/geroni/igad104.1067

**Published:** 2023-12-21

**Authors:** Ann Rhodes, Thomas Caprio, Tracey Gendron, Leland Waters

**Affiliations:** Virginia Commonwealth University, Richmond, Virginia, United States; University of Rochester, Rochester, New York, United States; Virginia Commonwealth University, Richmond, Virginia, United States; Virginia Commonwealth University, Richmond, Virginia, United States

## Abstract

The Special Focus Facility (SFF) program is an intensive, quality improvement intervention for 88 of the lowest-quality nursing facilities in 49 states. An additional 435 candidate facilities (SFFc) have similar quality problems but do not receive the SFF quality intervention. In the calendar year 2020, a total of 50 SFFs, and 197 SFFc were enrolled for all 12 months. These nursing facilities were compared on COVID-19 outcomes (Case Fatality Rate, staff cases, resident cases) to randomly selected 5-star nursing facilities in the same state for a total sample size of N=494. After controlling for case-mix hours per resident day resident (Case-Mix staffing) and size of nursing facility SFF and SFFc had significantly lower staff confirmed COVID-19 cases in 2020 as compared to 5-star facilities, adjusted R2 =.25, F (2, 4217)=358.2,p=<.005. Despite the lower staff COVID-19 rates, a higher resident Case Fatality Rate was observed, R2 =.013, F (4, 358) =15.42, p=<.005. Although both SFF and SFFc had lower total staff cases, there was a slightly higher rate of reported staff COVID-19 deaths in SFF and SFFc. However, this difference was not significant. R2 =.01, F (4, 4127)=12.32,p=.859. It is important to note that 2020 was the onset of the COVID-19 pandemic and, therefore, a time of extreme disruption from normalcy for residents and staff. However, these findings are a reminder that quality interventions should be designed to consider the linkages between staff and resident well-being and longevity in order to protect both residents and the workforce.

